# Age is just a number

**DOI:** 10.7554/eLife.34427

**Published:** 2018-01-24

**Authors:** Hiram Beltrán-Sánchez, Caleb Finch

**Affiliations:** 1 Department of Community Health Sciences and California Center for Population ResearchThe University of California Los AngelesLos AngelesUnited States; 2 Leonard Davis School of Gerontology and Dornsife CollegeUniversity of Southern CaliforniaLos AngelesUnited States

**Keywords:** naked mole-rat, H. glaber, Gompertz, aging, negligible senescence, Other

## Abstract

The naked mole rat defies the Gompertz law and shows no sign of increased mortality risk as it gets older.

**Related research article** Ruby JG, Smith M, Buffenstein R. 2018. Naked mole rat mortality rates defy Gompertzian laws by not increasing with age. *eLife* **7**:e31157. doi: 10.7554/eLife.31157

Bald and with wrinkly skin, the naked mole rat may not be the picture of youth, but these creatures continue to amaze us with their exceptionally long life. Naked mole rats are documented to live at least 30 years in captivity. But, they are not yet in the ranks of ‘gero-elites’: certain bats, for example, can live beyond 40 years (Podlutsky et al., 2005). Despite this longevity, naked mole rats show hardly any signs of getting older, such as problems with the heart, bones or metabolism. Females do not go through the menopause and continue to reproduce into their 30s. Even their cells have a remarkable resistance to oxidative damage (Edrey et al., 2011), and age-related chronic diseases, such as cancer, are rare.

So far, most of what we know about this animal is based on studies with small sample sizes, making it difficult to determine how long-lived they really are. Now, in eLife, J. Graham Ruby, Megan Smith and Rochelle Buffenstein of Calico Life Sciences report how naked mole rats never cease to surprise (Ruby et al., 2018).

A mathematical model called the Gompertz-Makeham law of mortality – which states that the risk of death increases exponentially with age – can be used to assess how long species live and what factors contribute to the mortality risk. Ruby et al. used this model to analyze an existing data set of 3,299 naked mole rats across a 30-year timespan and found that they did not conform to the Gompertz-Makeham law. In fact, their mortality hazard did not increase as they got older. This is unprecedented for mammals – one would not expect a small rodent such as the naked mole rat to live for more than six years, let alone show the first signs of aging at a time double its predicted maximum lifespan.

Previous studies suggest that aging nonetheless creeps in: naked mole rats can accumulate oxidative damage in their cells and tissues (a sign of aging) and experience muscle wasting, and there is also some evidence for cancer (Edrey et al., 2011; Andziak et al., 2006; Taylor et al., 2017). This motivates further consideration of Ruby et al.’s demographic criteria so that we can understand why their data show an absence of Gompertz mortality accelerations.

It can be assumed that when no deaths are observed in a group, there is still a risk of mortality – it may just be very low. However, when the number of deaths is low, errors in the sampling method could bias the estimates of the Gompertz parameters (Promislow et al., 1999). At its minimum measurable value, the death rate in a population is either 0 (no one dies) or 1 (a single individual dies). For the data studied by Ruby et al., this means that the minimum rate of mortality that can be accurately reported is 1/3,299: a baseline mortality of ~0.0003 per day. However, the baseline mortality reported in Ruby et al. is even lower (1/10,000 per day). This would indicate that their naked mole rat population is too small to correctly estimate the true mortality rate or the Gompertz parameters. In fact, sample sizes much larger than 3,299 would be needed to detect aging mortality acceleration.

Another component of the Gompertz-Makeham model is the rate of aging, from which the mortality rate doubling time (that is, the time required for the mortality rate to double) can be calculated. For example, assuming that the mortality rate begins to speed up when the naked mole rat reaches maturity at six months of age, the rate of Gompertz aging can be calculated to be about 0.006 per year of age. This is far lower than values found in modern human populations, where the rate ranges from 0.07 to 0.09 per year of age (Finch et al., 2014). Based on this, the mortality rate doubling time would be 115 years for the naked mole rat, compared to eight years for most human populations (Finch, 1998; Finch et al., 1990). This would suggest that unlike any other mammal, the naked mole rats have an extremely low rate of aging.

Its minimal age-related problems and long life-span make the naked mole rat an ideal candidate to study ‘negligible senescence’, a phenomenon that has been observed in a few species that reach advanced ages without increased mortality or disability (Finch, 2015). The study of Ruby et al. furthers our knowledge and provides a good framework for future studies to build upon.
